# Acute Renal Failure due to Leukaemic Infiltration in Chronic Lymphocytic Leukaemia

**DOI:** 10.1155/2015/469136

**Published:** 2015-06-04

**Authors:** Yusuf Kayar, Iskender Ekinci, Ilker Bay, Nuket Bayram Kayar, Jamshid Hamdard, Rumeyza Kazancıoğlu

**Affiliations:** ^1^Department of Gastroenterology, Bezmialem Vakif University, Vatan Street, Adnan Menderes Boulevard, 34093 Istanbul, Turkey; ^2^Department of Internal Medicine, Bezmialem Vakif University, Vatan Street, Adnan Menderes Boulevard, 34093 Istanbul, Turkey; ^3^Department of Internal Medicine, Istanbul Faculty of Medicine, Istanbul University, Istanbul, Turkey; ^4^Department of Family Medicine, Bagcilar Training and Research Hospital, Istanbul, Turkey; ^5^Department of Nephrology, Bezmialem Vakif University, Vatan Street, Adnan Menderes Boulevard, 34093 Istanbul, Turkey

## Abstract

Chronic lymphocytic leukemia (CLL) is a malignancy characterized by clonal proliferation and accumulation of B lymphocytes. Although leukaemic infiltration of the kidney is well recognized in CLL, acute renal failure (ARF) due to leukaemic infiltration is extremely rare. Here we present a case of ARF as the initial manifestation of CLL. The diagnosis was made by a kidney biopsy. Treatment with cyclophosphamide and prednisolone resulted in a completely improved renal function.

## 1. Introduction

Chronic lymphocytic leukemia (CLL) is a variety of chronic lymphoproliferative disorders. It is characterized by a progressive accumulation of monoclonal incompetent lymphocytes and is similar to peripheral B-cell neoplasm small lymphocytic lymphoma [1]. Although leukaemic infiltration of the kidney is relatively common in CLL, acute renal failure (ARF) due to leukaemic infiltration is extremely rare [2]. Herein, we report a patient who presented with ARF caused by leukaemic infiltration of the kidneys in previously nonexistent CLL and was treated with chemotherapy successfully.

## 2. Case Report

**Figure 1:**
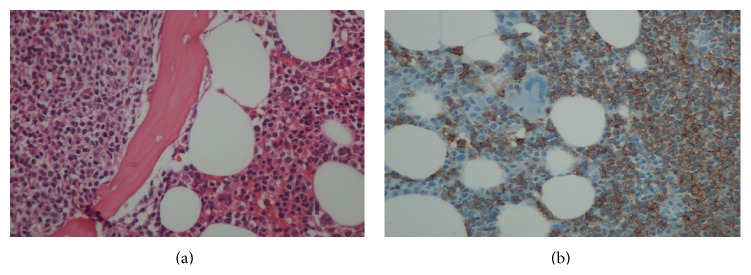
(a) Bone marrow biopsy revealed nodular paratrabecular and intertrabecular infiltration with small lymphocytes which appeared as mature cells (HE ×200). (b) Monoclonal B lymphocytes were positive for CD5, CD20, CD23, and CD79 in bone marrow (after the specimens were fixed in 10% formalin and embedded paraffin wax the immunohistochemical staining was performed using a Ventana immunostainer (Ventana Benchmark XT, ×200)).

**Figure 2:**
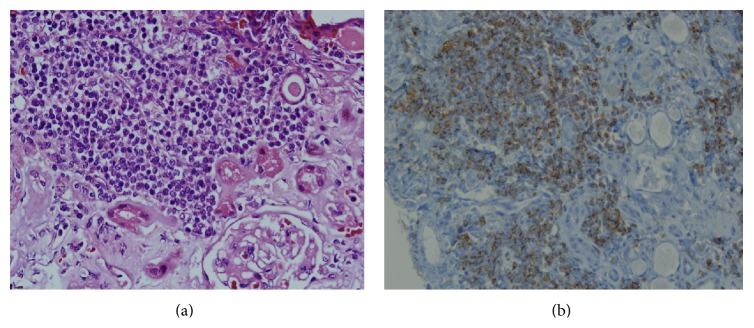
(a) Monotonous small lymphocytic infiltration in renal parenchyma (HE ×200). (b) Lymphocytic infiltration showing immunopositivity for CD5, CD20, and CD79 (after the specimens were fixed in 10% formalin and embedded paraffin wax the immunohistochemical staining was performed using a Ventana immunostainer (Ventana Benchmark XT, ×200)).

A 64-year-old man was admitted to our clinic with a 2-week history of nausea and vomiting. His past medical history included diabetes mellitus type 2 and hypertension controlled on metformin and irbesartan. He had no history of any renal disease. On physical examination the patient's blood pressure, pulse rate, respiratory rate, and body temperature were 110/70 mm Hg, 96 beats/minute, 14 breaths/minute, and 37°C, respectively. His physical examination also revealed splenomegaly with no evidence of hepatomegaly and lymphadenopathy. The fundus examination was normal. There were no other abnormal findings. Initial laboratory data showed the following values: leukocyte count was 18.6 × 10^9^/L (lymphocytes %65), hemoglobin 78 g/L, thrombocytes 265 × 10^9^/L, blood urea nitrogen 48 mg/dL, serum creatinine 2.2 mg/dL (estimated GFR: 38 mL/min/1.73 m^2^), sodium 137 mEq/L, potassium 3.2 mEq/L, lactate dehydrogenase 378 U/L, uric acid 4.5 mg/dL, albumin 3.7 g/dL, gamma globulin level 2.5 g/dL, and erythrocyte sedimentation rate 51 mm/h. Serology was negative for antinuclear antibody, antineutrophil cytoplasmic antibody (ANCA), antiglomerular basement membrane antibody, HBsAg, and anti-HCV. Serum complement (C3 and C4) levels were normal and cryoglobulin was negative. Both monoclonal immunoglobulin levels and free light chain analysis for kappa and lambda in serum and urine were normal. Urine analysis showed only mild proteinuria (24 h protein excretion was 100 mg) without dysmorphic cells or casts. Blood smear revealed mild mature lymphocytosis. Sinus rhythm was observed on electrocardiogram. An abdominal ultrasonography (USG) revealed normal kidney size. A bone marrow biopsy was consistent with CLL and these monoclonal B lymphocytes were determined by means of immunological markers: CD5, CD20, CD23, and CD79 (Figures 1(a) and 1(b)). The cause of the ARF was not apparent despite numerous investigations. A fine needle aspiration of the kidney was performed, and it was suggestive of diffuse leukaemic infiltration of the renal parenchyma (Figure 2(a)). Immunohistochemistry showed these cells were positive for CD20, CD5, and CD79 (Figure 2(b)). There was no evidence of amyloid and light chain deposition. Morphological and immunohistochemical findings supported a diagnosis of ARF due to leukaemic infiltration of the kidneys in CLL. The patient was started on a combination of cyclophosphamide (50 mg/day) and methylprednisolone (48 mg/day). The followed parameters on admission and during treatment were compiled in Table 1. The patient completely recovered from renal dysfunction a month after the initiation of therapy.

## 3. Discussion

Chronic lymphocytic leukemia (CLL) is a neoplastic condition of B-cells, and it usually affects the lymph nodes, liver, spleen, and bone marrow [2]. Renal infiltration by CLL cells has been documented in about 63% to 90% of all CLL patients who underwent a postmortem autopsy, but it is seldom associated with renal failure [3, 4]. In a large retrospective study in 700 patients with non-Hodgkin's lymphoma and CLL, only 3 of 17 patients with renal involvement in CLL were directly related to CLL [5]. Here we report a case of ARF caused by CLL cells' infiltration which was treated successfully with a combination therapy of cyclophosphamide and methylprednisolone.

Acute renal failure in CLL patients can be associated with acute tubular necrosis, uric acid nephropathy, light chain nephropathy, obstructive nephropathy, amyloidosis, hypercalcemia, glomerulonephritis, and cryoglobulinemia [6]. Although the mechanism of renal failure in CLL is not clear it may be associated with intrarenal obstruction and ischemia which occurs secondary to the compression of the tubular lumen with CLL cells [7]. Renal involvement by CLL cells could be nodular or diffuse and may cause fibrosis in the same areas. Kidney size can be either increased or normal and proteinuria has been generally mild [2]. In the present case, prerenal failure, obstructive uropathy due to stones or urinary tract compression of the intra-abdominal lymphadenopathy, use of nephrotoxic agents, tumor lysis syndrome, and infectious disease were excluded for differential diagnosis. There were no signs to suggest nephrotic syndrome in our patient. The kidney size was normal with mild proteinuria. A fine needle aspiration biopsy of the kidney was performed to confirm or rule out a glomerulonephritis. It was suggestive of leukaemic infiltration of the renal parenchyma. The infiltrating lymphocytes were CD20^+^, CD5^+^, and CD79^+^ in immunochemical analysis.

The treatment options include steroids, chlorambucil, vincristine, cyclophosphamide, rituximab, fludarabine, and irradiation of the kidneys or a combination therapy consisting of these agents. The response is variable from complete response to a partial or no response [8].

In general, the preferred treatment modality for CLL is RFC (rituximab + fludarabine + cyclophosphamide) protocol in our clinic. Rituximab could not be used because of reimbursement issues regarding this drug in these patients as an initial treatment in our country. Use of fludarabine was contraindicated in our patient because of Coombs positive hemolytic anemia. Hence, a combination of cyclophosphamide and methylprednisolone was preferred in our patient and the patient responded very well with regression of renal dysfunction and an improvement in the peripheral lymphocyte count, meaning complete remission.

From our case report and the other few cases reported in the literature, we suggest that leukaemic infiltration in the kidneys should be kept in mind when a patient with CLL presents with ARF. Acute renal failure can also occur as the first presentation of CLL like the present case. A kidney biopsy is required for diagnosis. Keeping this possibility in mind is important because it responds very well to chemotherapy, as in our case treated successfully with cyclophosphamide and methylprednisolone.

## Figures and Tables

**Table 1:** The laboratory data on admission and during treatment.

Parameter	Admission	7th day of treatment	15th day of treatment	21st day of treatment	At the end of treatment
Blood pressure (mmHg)	110/70	115/75	120/80	115/75	120/70
Urine volume/24 hours (cc)	1200	1100	1400	1350	1300
Body weight (kg)	60	61	62	61	61
Peripheral lymphocyte count (10^9^/L)	18.6	17.9	16.2	14.3	12.2
Glucose (mg/dL)	98	93	95	101	90
Creatinine (mg/dL)	2.2	2.5	1.9	1.5	1.2
Sodium (mEq/L)	137	139	135	137	140
Potassium (mEq/L)	3.2	3.8	3.7	3.8	4.1
Calcium (mg/dL)	8.7	9.5	9.2	9.3	8.9
Phosphate (mg/dL)	3.1	3.7	3.3	3.5	2.9
Albumin (g/dL)	3.2	3.5	3.4	3.7	3.8
Uric acid (mg/dL)	4.5	4.4	4.2	4.6	4.7
